# Plant-Produced Recombinant Influenza A Virus Candidate Vaccine Based on Flagellin Linked to Conservative Fragments of M2 Protein and Hemagglutintin

**DOI:** 10.3390/plants9020162

**Published:** 2020-01-29

**Authors:** Elena A. Blokhina, Eugenia S. Mardanova, Liudmila A. Stepanova, Liudmila M. Tsybalova, Nikolai V. Ravin

**Affiliations:** 1 Institute of Bioengineering, Research Center of Biotechnology of the Russian Academy of Sciences, Moscow 101000, Russia; blohina-lena87@mail.ru (E.A.B.); mardanovaes@mail.ru (E.S.M.); 2 Research Institute of Influenza, Russian Ministry of Health, St. Petersburg 23805, Russia; stepanoval60@mail.ru (L.A.S.); sovet@influenza.spb.ru (L.M.T.)

**Keywords:** influenza, plant, vaccine, flagellin, M2e peptide, hemagglutinin

## Abstract

The development of recombinant influenza vaccines with broad spectrum protection is an important task. The combination of conservative viral antigens, such as M2e, the extracellular domain of the transmembrane protein M2, and conserved regions of the second subunit of hemagglutinin (HA), provides an opportunity for the development of universal influenza vaccines. Immunogenicity of the antigens could be enhanced by fusion to bacterial flagellin, the ligand for Toll-like receptor 5, acting as a powerful mucosal adjuvant. In this study, we report the transient expression in plants of a recombinant protein comprising flagellin of *Salmonella typhimurium* fused to the conserved region of the second subunit of HA (76–130 a.a.) of the first phylogenetic group of influenza A viruses and four tandem copies of the M2e peptide. The hybrid protein was expressed in *Nicotiana benthamiana* plants using the self-replicating potato virus X-based vector pEff up to 300 µg/g of fresh leaf tissue. The intranasal immunization of mice with purified fusion protein induced high levels of M2e-specific serum antibodies and provided protection against lethal challenge with influenza A virus strain A/Aichi/2/68(H3N2). Our results show that M2e and hemagglutinin-derived peptide can be used as important targets for the development of a plant-produced vaccine against influenza.

## 1. Introduction

Influenza is a widely distributed viral infection of humans and animals. Traditional influenza vaccines are strain–specific and have limited efficiency in the prevention of infections caused by newly emerging strains of influenza viruses [1]. The development of recombinant influenza vaccines with a broad protection spectrum and short production time is an important task. Such universal vaccines are based on the use of conserved viral antigens [2,3,4]. The design of a candidate vaccine protein composed of two or more conserved target antigens that could induce different arms of immune response (antibodies with different modes of action, CD4+ and CD8+ T-lymphocytes etc) would boost the efficacy of such protein-based vaccines.

Hemagglutinin (HA) is the main antigen of the influenza virus; it induces the formation of virus-neutralizing antibodies during immunization. The variability of the HA is responsible for the antigenic variability of the influenza virus. The HA protein represents an attractive target for vaccine development because of its important roles in the early stages of virus infection. Although the HA protein is highly variable, its second (HA2) subunit, forming a stalk domain, is relatively conserved [5], especially within the particular phylogenetic group of the HA proteins (the first group-H1, H2, H5, H6, H8, H9, H11, H12, H13, and H16 subtypes, the second group-H3, H4, H7, H10, H14, and H15 subtypes), and can be used as a candidate vaccine antigen. HA2-based recombinant proteins have been shown to induce humoral and T-cell immune responses and provide protection against infection [6,7]. 

M2e, the extracellular domain of the transmembrane protein M2 of influenza A virus is one of the most promising candidates for the development of “universal” vaccine since its sequence has been virtually unchanged in all human isolates since 1933, and in strains of animal origin it differs in only a few amino acids [8,9]. M2e is a short and poorly immunogenic peptide; however, when fused to an adjuvant or carrier virus-like particle it becomes highly immunogenic. A number of candidate vaccines based on M2e were able to induce a strong M2e-specific humoral immune response to protect animals against influenza A virus challenge [9,10]. A combination of M2e and HA2 in a single vaccine protein is a promising approach for the development of influenza vaccines with a broad spectrum of protection [11,12,13].

However, by themselves, both M2e and HA2 are poorly immunogenic and neither immunization with traditional vaccines nor influenza infection stimulates the production of significant amounts of antibodies to these peptides [14,15,16]. Immunogenicity of these antigens could be enhanced by their fusion to highly immunogenic adjuvants or protein carriers [9,17]. One of the most promising adjuvants is the bacterial flagellin, a ligand of Toll-like receptor (TLR) 5. TLRs play important roles in controlling the adaptive immune response by arming dendritic cells, triggering important costimulatory and regulatory mechanisms, and facilitating the presentation of antigens to the immune system [18]. Attachment of the antigen to flagellin can significantly increase its immunogenicity and lead to the formation of a protective immune response that has been demonstrated for various infections including influenza [19,20,21]. Notably, flagellin is an efficient mucosal adjuvant and can be used for non-invasive intranasal administration of vaccines. 

Plants could become promising biofactories for expression of recombinant proteins due to the low final cost and inherent safety of products resulting from the absence of pathogens common to plants and animals. Previously, we reported the transient expression in plants of recombinant protein Flg-4M comprising flagellin FljB of *Salmonella typhimurium* fused to four tandem copies of the M2e peptide, namely two copies of human consensus M2e sequence, and two copies of the M2e peptide of avian influenza virus strain A/Chicken/Kurgan/05/2005 (H5N1). The use of a self-replicating recombinant viral vector based on the potato virus X (PVX) allowed for the expression of Flg-4M in *Nicotiana benthaminana* leaves at a very high level, about 1 mg/g of fresh leaf tissue. Intranasal immunization of mice with this candidate vaccine induced high levels of M2e-specific serum antibodies and provided protection against lethal challenge with different strains of influenza virus [22]. However, unlike antibodies to hemagglutinin, antibodies specific to M2e cannot directly neutralize the virus.

The goal of this study was to develop a plant-produced candidate influenza vaccine based on the combination of two conserved influenza antigens (M2e and HA2) and to investigate the immune response and protective activity in an animal model. In this work we combined the advantages of a highly efficient PVX-based expression system, M2e and HA2 influenza A antigens and adjuvant properties of the bacterial flagellin to develop a new vaccine candidate.

## 2. Results

### 2.1. Viral Vectors for the Expression of Flagellin Linked to the HA2-1 Peptide and Four Copies of M2e in N. Benthamiana Plants

**Figure 1:**
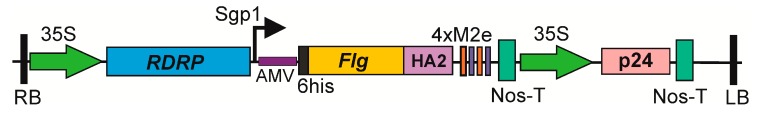
Structure of the expression vector pEff_Flg4M2eHA2-1. RDRP, RNA-dependent RNA polymerase of PVX; Sgp1, the first promoter of the subgenomic RNA of PXV; AMV, the leader sequence of RNA 4 of alfalfa mosaic virus; 6his, sequence that encodes the N-terminal 6-histidine tag; Flg, *S. typhimurium* flagellin; HA2, sequence of the HA fragment (76–130 a.a.); 4M2e, the sequence of four tandem copies of M2e peptide arranged as M2eh-M2es-M2eh-M2es; 35S, promoter of the cauliflower mosaic virus RNA; NosT, terminator of the nopaline synthase gene from *Agrobacterium tumefaciens*; P24, gene of silencing suppressor from grapevine leafroll-associated virus-2; LB and RB are the left and right borders of T-DNA.

A fusion protein, Flg4M2eHA2-1, containing two copies of the human consensus M2e sequence (M2eh) and two copies of M2e of the pandemic influenza virus strain A/H1N1pdm09 (M2es), fused to the C-terminus of S. typhimurium FljB was designed (Figure 1). Individual copies of the M2e peptides were separated by glycine-rich GGGSG linkers to facilitate folding of the fusion protein. A 6-histidine tag was attached to the N-terminus of the hybrid protein to facilitate their purification by metal affinity chromatography. The consensus sequence of a highly conserved alpha helical region (from 76 to 130 a.a.) of the second subunit of HA of influenza A viruses of the first phylogenetic group was inserted between flagellin and 4M2e sequences (Figure 1). The fusion protein consisted of 686 a.a. and had a calculated molecular mass of 72.6 kD. Flagellin without M2e and HA2-1 fusions was used as a control.

The pEff viral vector [23] was used to express the target proteins in N. benthamiana plants. This expression vector is based on the genome of PVX and includes the 5’-untranslated region of the PVX genome, the RNA-dependent RNA polymerase gene, the first promoter of the subgenomic RNA, the 5’-untranslated region of the alfalfa mosaic virus (AMV) RNA 4, acting as a translational enhancer, the green fluorescent protein gene (gfp), the last 60 nucleotides of the coat protein gene of PVX, and the 3’-untranslated region of the PVX genome. The entire expression cassette was placed between the 35S promoter and the NosT terminator and cloned within the tDNA region of the binary vector that can replicate in E. coli and A. tumefaciens cells. The genes coding for flagellin and a fusion protein, Flg4M2eHA2-1, were cloned into pEff, replacing the gfp gene to create recombinant viral vectors. The pEff vector additionally contains an expression cassette for the silencing suppressor, P24, from grapevine leafroll-associated virus-2 to suppress post-transcriptional gene silencing in plant cells (Figure 1).

### 2.2. Expression of Recombinant Proteins in N. benthamiana Plants

To express the Flg4M2eHA2-1 protein, the corresponding vector was inserted into *A. tumefaciens* strain GV3101, which was used for agroinfiltration of leaves of *N. benthamiana*. After agroinfiltration, the vector tDNA was transferred from the agrobacterium to the plant cells. As a result of transcription from the 35S promoter in infected cells, the RNA of the viral vector became synthesized, its translation and replication occurred, and the subgenomic RNA encoding the target gene and the protein product became synthesized. 

Total protein was isolated at 2, 4, 6, and 8 days after agroinfiltration and analyzed using SDS-PAGE and Western blotting. The level of expression of Flg4M2eHA2-1 reached its maximum on the fourth day (Figure S1). An analysis of the total protein sample and the soluble fraction showed that Flg4M2eHA2-1 was almost completely insoluble and therefore should be purified under denaturing conditions.

For the scaled-up production of Flg4M2eHA2-1, the protein samples were isolated from agroinfiltrated leaves 4 days after infection. Recombinant protein was isolated from 80 g of leaves using metal-affinity chromatography under denaturing conditions. After purification the protein samples were dialyzed against phosphate-buffered saline (PBS). The obtained protein remained soluble and was subjected to ultracentrifugation to remove RuBisCo aggregates.

**Figure 2:**
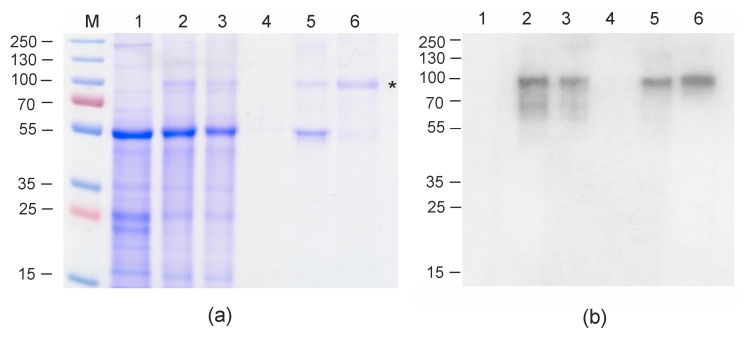
Coomassie brilliant blue stained gel (**a**) and western blot (**b**) of proteins isolated from *N. benthamiana* plants and separated by SDS-PAGE. M, molecular weight marker (kD); lane 1, total proteins from empty leaves; lane 2, total protein from leaf infiltrated with vector pEff_Flg4M2eHA2-1; lane 3, protein purification on Ni-NTA column: flow-through (unbound) fraction; lane 4, protein purification on Ni-NTA column: washed fraction; lane 5, protein purification on Ni-NTA column: eluate; lane 6, purified Flg4M2eHA2-1 protein after ultracentrifugation (position shown by an asterisk). Positions and sizes of molecular weight markers (kD) are shown in (b) to the left of the image.

Protein samples were analyzed using SDS-PAGE and Western blotting with M2e-specific antibodies. The data presented in Figure 2 shows that the Flg4M2eHA2-1 protein was successfully produced and the level of expression was about 300 µg/g of fresh leaf tissue. The yield of Flg4M2eHA2-1 after purification was 60 μg per 1 g of green leaf biomass; about 5 mg of purified protein was obtained and used for animal experiments. Similar expression levels of M2e-containing proteins in plants have been achieved in other studies [24,25,26]. These values were lower than those obtained in the expression of flagellin with four attached copies of the M2e of the avian influenza virus (0.5–1 mg/g before purification [23]), but they were sufficient for the production of recombinant protein quantities necessary for immunogenicity testing and estimation of protective action in animal studies.

### 2.3. Immunogenicity and Protective Activity of Plant-Produced Flg4M2eHA2-1 Protein

To characterize the immunogenicity and protective action of the candidate vaccine, mice were immunized thrice with the plant-produced Flg4M2eHA2-1 and an empty flagellin as a control. Mice were vaccinated intranasally three times at two-week intervals with 10 μg of protein without additional adjuvants. Blood and broncho-alveolar lavage (BAL) samples were taken after the third immunization.

**Figure 3:**
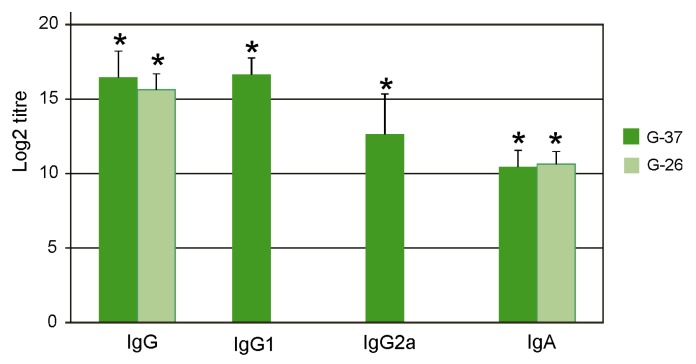
Titers of IgG, IgG1, IgG2a, and IgA antibodies to synthetic M2e peptides G-37 and G-26 in sera of immunized mice after the third immunization. The results are expressed as the mean titer ± the standard deviation for each group of five mice expressed in log2. Note that the titers of IgG1 and IgG2a antibodies to G-26 peptide were not measured. Titers lower than 400 were detected for mice immunized with PBS or Flg. Significant differences (*p* < 0.05) from control groups are marked with an asterisk (*).

The sera were analyzed by ELISA to identify antibodies directed against M2e. IgG, IgG1, IgG2a, and IgA antibodies were detected by ELISA using plates coated with synthetic peptides G-37 and G-26, whose sequences corresponded to M2eh and M2es, respectively. It was found that immunization with Flg4M2eHA2-1 induced high titers of the M2e-specific IgG in serum, predominantly of the IgG1 subtype (Figure 3). The difference between titers of IgG1 and IgG2a was significant (*p* < 0.01). Induced IgG and IgA antibodies nearly equally efficiently bound to peptides G-37 and G-26.

**Figure 4:**
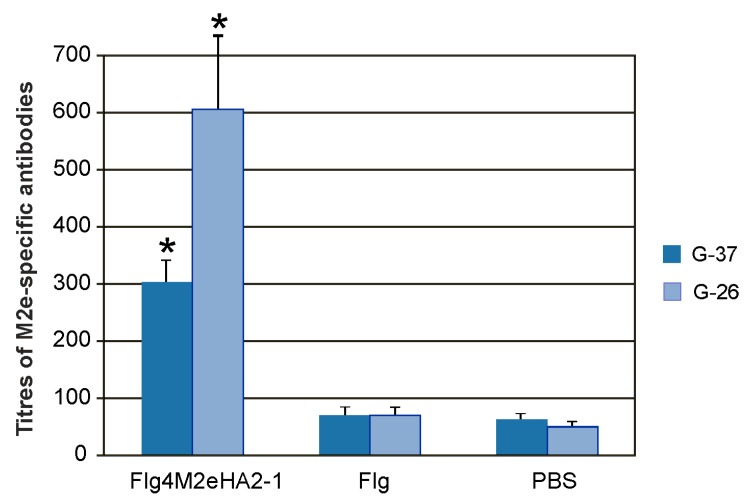
Titers of IgG antibodies to synthetic M2e peptides G-26 and G-37 in BALs of immunized mice after the third immunization. The results are expressed as the mean titer ± the standard deviation for each group. Significant differences (*p* < 0.05) from control groups are marked with an asterisk (*). The differences between titers of M2e-specific antibodies recognizing G-26 and G-37 were not statistically significant.

To investigate IgA and IgG responses in mucosal secretions, titers of M2e-specific antibodies were determined in the BALs. Intranasal immunization with the Flg4M2eHA2-1 fusion protein induced significantly higher M2e-specific IgG antibody titers in BALs compared to the PBS or Flg preparation (Figure 4), while no statistically significant increase in the level of M2e-specific IgA antibody titers was detected.

**Figure 5:**
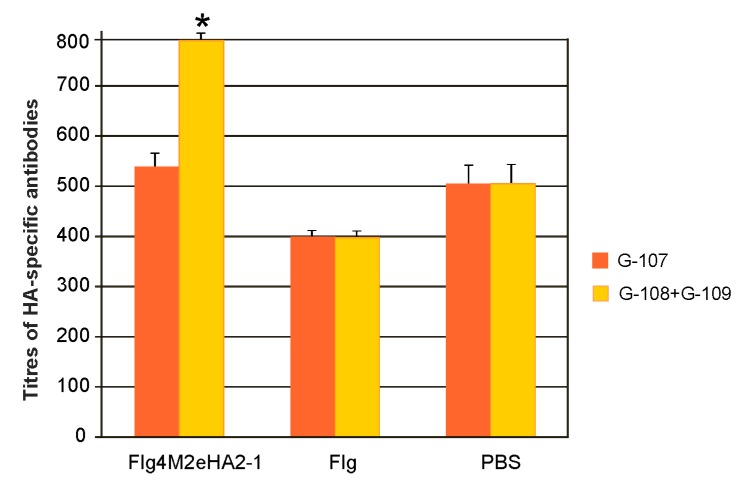
Titers of IgG antibodies to synthetic HA2 peptides in sera of immunized mice after the third immunization. The results are expressed as the mean titer ± the standard deviation for each group. Significant differences (*p* < 0.05) from control groups are marked with an asterisk (*). The differences between titers of M2e-specific antibodies recognizing G-107 and G-108+G-109 were not statistically significant.

Serum IgG antibodies against HA2 were identified by ELISA using plates coated with the synthetic peptide G-107 or a mixture of G-108 and G109. The G-107 peptide represented the consensus sequence of a fragment from 93 to 122 a.a. of HA2 of influenza A viruses of the first phylogenetic group, and it was identical in sequence to a part of HA2-1 (from 76 to 130 a.a. of HA2). Peptides G-108 and G109 correspond to the 99–130 a.a. and 76–106 a.a. regions of HA2 of the second phylogenetic group of influenza A. We found that immunization with Flg4M2eHA2-1 induced rather low titers of the HA-specific IgG (Figure 5). The induced antibodies bound more efficiently to a mixture of G-108 and G109 peptides, and the differences in antibody titers compared to the Flg and PBS controls were significant (*p* < 0.05).

**Figure 6:**
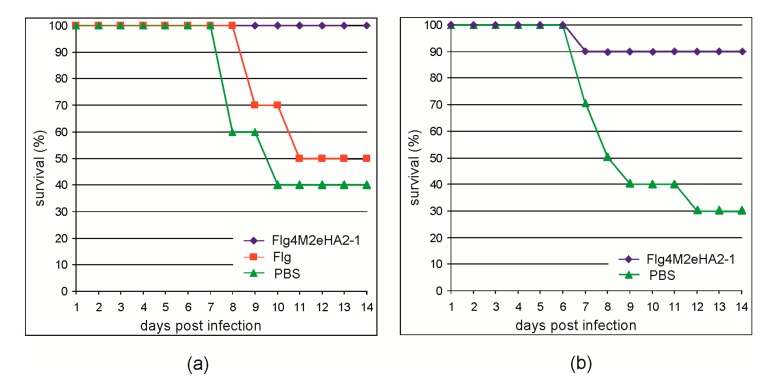
Protective efficiency of the plant-produced Flg4M2eHA2-1 protein. Mice were challenged with 2LD_50_ (**a**) or 5LD_50_ (**b**) of A/Aichi/2/68 (H3N2) influenza A virus. The survival of immunized and control mice was monitored for 14 days post-infection.

To evaluate the protective action of the Flg4M2eHA2-1 protein, the immunized mice were challenged with mouse-adapted influenza A strain A/Aichi/2/68 (H3N2) virus. As shown in Figure 6, all mice immunized with Flg4M2eHA2-1 survived the challenge with the 2xLD_50_ dose, while the rate of survival among the control groups was significantly lower. In the second experiment, 90% of mice immunized with Flg4M2eHA2-1 survived the 5xLD_50_ lethal challenge, while the rate of survival among the control mice was 30%.

We observed a decrease in lung viral titers following the challenge of immunized mice with 5xLD_50_ of strain A/Aichi/2/68 (H3N2) as compared to control mice. Vaccination limited the replication of the virus in the lungs decreasing its titer from 4.3 to 3.2 log_10_ TCID_50_, this difference was significant (*p* < 0.05).

## 3. Discussion

The purpose of the present work was to develop a plant-produced candidate influenza vaccine based on the M2e peptide and conservative fragment of the second subunit of HA, linked to bacterial flagellin, acting as an adjuvant for mucosal immunization. The inclusion of both antigens in vaccine preparation simultaneously can provide a more diverse spectrum of antibodies after immunization and thus increase the efficiency of immunization [27,28]. In order to increase the broadness of the vaccine, we used four tandem copies of M2e—two copies of the “consensus” M2e peptide of human influenza A viruses and two copies of the M2e peptide of the pandemic strain A/H1N1pdm09. These sequences differ in four of the 23 positions, and these differences are important for the specificity of the immune response [29]. Since immunization with the Flg4M2eHA2-1 protein induced a high level of IgG antibodies recognizing both types of M2e, it could be expected that the new candidate vaccine will provide protection against both typical human influenza strains and A/H1N1pdm09, although we were not able to assess the latter strain.

HA is the main antigen of the influenza virus. In contrast to M2e, it induces the formation of virus-neutralizing antibodies during immunization. The high variability of the HA is responsible for the antigenic variability of the influenza virus but the second subunit (HA2) is more conservative [30]. Several candidate vaccines, including “consensus” HA2 fragments of the first and the second phylogenetic group have been described in recent years [6,31]. In this study we used the highly conserved region from 76 to 130 a.a. of HA2, which can provide protective properties in vivo [6]. Since the HA2 peptides are located in the “internal” part of the HA, it was included in the hybrid protein between the flagellin and the tandem copies of M2e. However, immunization with Flg4M2eHA2-1 induced only a low level of antibodies capable of binding with HA2 synthetic peptides, and most likely anti-HA2 IgG in this vaccine design does not have a major role in protection. A similar result involving a strong anti-M2e immune response and a weak response to HA2 was reported for a similar candidate vaccine based on four copies of M2e and HA2 peptide from phylogenetic group 2 of influenza viruses linked to flagellin [13].

Although there are numerous successful examples of the expression of recombinant proteins—including influenza vaccine candidate—in plants [4,32], the yield of the recombinant protein strongly depends on its properties and the efficiency of the expression system. Agroinfiltration of plant leaves with a self-replicating plant viral vector is a rapid and efficient method for protein production in plants. In this study, we used PVX-based vector pEff for the expression of recombinant proteins in plant cells. This vector enabled a very high level of expression of the target proteins of up to 1 mg/g of fresh leaf tissue [24]. Recently, we employed a similar expression system to produce in *N. benthaminana* plants a recombinant protein, Flg-4M, comprising flagellin fused to four copies of the M2e peptide, representing the human consensus M2e sequence and the M2e peptide of avian influenza strain A/Chicken/Kurgan/05/2005. The protein was produced at a level of about 1 mg/g of fresh leaf tissue, and it appeared to be in the soluble fraction of the proteins extracted from the leaves [23]. Therefore, it likely that inclusion of the HA2 peptide in the Flg4M2eHA2-1 vaccine candidate in this work resulted in lower expression and insolubility of the hybrid protein. A high number of hydrophobic amino acid residues in HA2-1 (21 out of 55 a.a.) probably explains the insolubility of the Flg4M2eHA2-1 protein. 

Intranasal immunization of mice with the plant-produced Flg4M2eHA2-1 protein provided protection against the lethal influenza infection. Complete protection was observed in an experiment where immunized mice were challenged with 2xLD_50_ of A/Aichi/2/68 (H3N2) and 90% protection in the case of a higher infection doze of 5xLD_50_. The protection strongly depended on the presence of influenza antigens in the hybrid protein, since immunization with plant-produced empty flagellin conferred no extra protection relative to the PBS-immunized mice. Incomplete protection could be related to the low immunization dose (only 10 μg) and the method of vaccine delivery. Protection efficiencies between 80% and 100% were previously observed upon 5xLD_50_ challenge of immunized mice by different influenza strains in the case of plant-produced flagellin fused to four copies of M2e [23]. 

## 4. Materials and methods 

### 4.1. Bacterial Strains, Media and Reagents

*Escherichia coli* bacteria were grown in LB broth or on Petri dishes with LB agar at 37 °C, and the *Agrobacterium tumefaciens* strain GV3101 was grown at 28 °C. If necessary, the media were supplemented with the following antibiotics: kanamycin (50 μg/mL), rifampicin (50 μg/mL), or gentamycin (25 μg/mL).

### 4.2. Antigens of the Influenza Virus, Synthetic Nucleotide Sequences and Expression Vector

The “consensus” M2e sequence of human influenza A viruses (M2eh, SLLTEVETPIRNEWGCRCNDSSD [9]), the M2e sequence of the pandemic influenza strain A/H1N1pdm09 (M2es, SLLTEVETPTRSEWECRCSDSSD), and the consensus sequence of the HA2 region (from 76 to 130 a.a.) of influenza A viruses of the first phylogenetic group (HA2-1, RLENLNKKMEDGFLDVWTYNAELLVLMENERTLDFHDSNVKNLYDKVRMQLRDNA) were used. Nucleotide sequences encoding HA2-1 and a fusion of M2eh-M2es-M2eh-M2es peptides were synthesized in vitro.

The target gene encoding the flagellin sequence fused to the HA2-1 fragment followed by four tandem copies of M2e, arranged in the order M2eh-M2es-M2eh-M2es, was cloned into the pEff vector [24] as described previously [33]. The recombinant vector pEff_Flg4M2eHA2-1 was transferred from *E. coli* to the *A. tumefaciens* GV3101 strain using electroporation.

### 4.3. Agroinfiltration of N. benthamiana Plants

Plants were grown in a greenhouse with additional illumination (full-spectrum phytolamp, 16 h light/day photoperiod) at 28 °C until the formation of 5–6 true leaves. Agrobacteria carrying recombinant expression vectors were grown overnight with shaking at 28 °C. The cells (1.5 mL) were harvested by centrifugation for 5 min at 4000 g, and the pellet was resuspended in 1.5 mL of buffer containing 10 mM MES (pH 5.5) and 10 mM MgSO_4_. Leaves of *N. benthamiana* plants were infiltrated with suspension of agrobacteria (OD_600_ ~0.2) using a syringe without a needle. After agroinfiltration plants were grown in a greenhouse under the same conditions.

### 4.4. Isolation of Recombinant Proteins from Plant Tissue

The plant-produced Flg4M2eHA2-1 protein carrying the N-terminal 6-histidine tag was isolated under denaturing conditions on Ni-NTA resin (Promega, Madison, WI, USA). Four days after infiltration, the *N. benthamiana* leaves were powdered to homogeneity in liquid nitrogen. A solution containing 6 M guanidine HCI, 50 mM NaH_2_PO_4_, 500 mM NaCI, and 5 mM imidazole, (pH 8.0) was added to the powdered plant tissue. The resulting mixture was centrifuged at 14,000 *g* for 15 min, and the supernatant was applied to Ni-NTA resin equilibrated with the same buffer and incubated for 60 min. Then, the resin was washed twice with the same buffer. The recombinant protein Flg4M2eHA2-1 was eluted with buffer containing 4 M urea, 50 mM NaH_2_PO_4_, 300 mM NaCI, and 500 mM imidazole. After elution, the protein was dialyzed against PBS (1:100, three changes of buffer) using Slide-A-Lyzer Mini dialysis devices (Thermo Fisher Scientific, Waltham, MA, USA). Dialyzed protein preparation was ultracentrifuged at 35,000 rpm (rotor SW40Ti, Optima L-90K centrifuge, Beckman Coulter) at 15 °C for 6 h and then the supernatant was stored at −20 °C. This stage enabled efficient removal of RuBisCo aggregates (Figure 2). Proteins were quantitated by a Bradford assay (Bio-Rad, Waltham, MA, USA), following the manufacturer’s instructions.

The control recombinant flagellin was expressed in *N. benthamiana* and purified as described previously [23].

### 4.5. SDS-PAGE and Western-Blotting of Protein Preparations

The leaf tissue in the agroinfiltrated area was homogenized with a mortar and pestle and mixed with an equal volume of 2× SDS-PAGE loading buffer (20% glycerol, 5% SDS, 62.5 mM Tris-HCI pH 6.8, 0.5% bromphenol blue, 5% β-mercaptoethanol). The purified proteins were diluted two-fold in the same buffer. The samples were boiled for 10 min and subjected to SDS-PAGE in a 10% (w/v) gel. After electrophoresis, the gel was either stained with Coomassie brilliant blue or the proteins were transferred from the gel onto a Hybond-P membrane (GE Healthcare, Chicago, IL, USA) by electroblotting. To prevent the nonspecific binding of antibodies with the membrane, it was treated with a 5% (*w/v*) solution of dry milk in TBS-T (150 mM NaCI, 20 mM Tris, 0.1 % Tween 20, pH 8.0) buffer. The membrane was probed with mouse monoclonal antibodies specific for the M2e peptide (Anti-Influenza A Virus M2 Protein antibody [14C2] (ab5416); Abcam, Cambridge, UK) and then incubated with Anti-Mouse IgG HRP Conjugate (W4021, Promega). Specific protein complexes were detected using a Western Blot ECL Plus kit (GE Healthcare).

### 4.6. Mouse Immunization

Female BALB/c mice (16–18 g) were vaccinated intranasally three times at two-week intervals with 10 μg (0.5 mg/mL in PBS, 10 μL per nostril) of recombinant protein (Flg4M2eHA2-1 or Flg) without additional adjuvants. Control mice intranasally received 20 μL of PBS.

### 4.7. Synthetic Peptides

The following synthetic peptides were used in the ELISA experiments: G37, human “consensus” M2e sequence (M2eh); G-26, M2e of influenza strain A/H1N1pdm09 (M2es); G-107, amino acids 93–122 of HA2 of the first phylogenetic group of influenza A viruses (TYNAELLVLMENERTLDFHDSNVKNLYDKV); G-108, amino acids 99–130 of HA2 of the second phylogenetic group of influenza A viruses (LVALENQHTIDLTDSEMNKLFEKTRRQLRENA); G-109, amino acids 76-106 of HA2 of the second phylogenetic group of influenza A viruses (RIQDLEKYVEDTKIDLWSYNAELLVALENQH). 

### 4.8. Antibody Detection by ELISA

Antigen-specific levels of antibodies were determined by ELISA performed in 96-well microtiter plates coated overnight at 4 °C with the synthetic peptide (5 μg/mL) in PBS (pH 7.2). The plates were blocked with PBS containing 5% fetal calf serum (FCS) for 1 h at room temperature. The plates were washed 3 times in PBS containing Tween. The diluted samples were added in volumes of 100 μL per well and the plates were incubated for 1 hour at room temperature. As a conjugate, HRP-labelled goat anti-mouse IgG (ab97040, Abcam), IgG1 (ab98693, Abcam), IgG2a (ab98698, Abcam), and IgA (ab97235, Abcam) were used at 1:5000 to 1:20,000 dilutions. After adding tetramethylbenzidine substrate (BD Bioscience) and monitoring the color development, the reaction was stopped withy H_2_SO_4_, and the OD at 450 nm was measured on a microplate spectrophotometer.

### 4.9. Obtaining Broncho-Alveolar Lavages (BALs)

Five mice from each group were sacrificed by placing into the CO_2_-box for euthanasia (Vet Tech Solutions) after the third immunization. The lungs were removed aseptically and homogenized in 2.7 mL PBS using a Tissue Lyser II homogenizer (Qiagen, Hilden, Germany) to achieve 10% (w/v) suspensions, and they were centrifuged (15 min, 3000 rpm, 4°C) to remove cellular debris before storage at −20 °C.

### 4.10. Influenza Viruses and Challenge

Mouse-adapted A/Aichi/2/68 (H3N2) influenza A virus obtained from the Collection of Influenza and Acute Respiratory Viruses at the Research Institute of Influenza was used to challenge immunized mice (15 in each group) at doses of 2xLD_50_ and 5xLD_50_. The LD_50_ was calculated using the Reed-Muench method [34]. The virus was administered intranasally in a total volume of 20 μL (10 μL per nostril) to mice anaesthetized by ether. Ten mice from each group were monitored daily for the survival rate following the viral challenge for a period of 2 weeks. Experimental work with influenza strains was carried in a BSL2 facility.

### 4.11. Lung Virus Titers

Five mice from each group were sacrificed (CO_2_-chamber, as described above) on day 6 post-infection, and the lungs were removed. The viral titer in the lungs was determined as described previously [13] and expressed as the log 50% tissue culture infectious dose (TCID_50_).

### 4.12. Statistical Analysis

The differences between antibody levels and viral titers in lung suspensions were evaluated by the Mann–Whitney U-test. Significant differences in survival among mouse groups were analyzed by the Mantel-Cox test. If a *p* value was less than 0.05, the difference was considered to be significant.

### 4.13. Ethics Statement

The study was carried out in accordance with the Russian Guidelines for the Care and Use of Laboratory Animals. The protocol was approved by the Committee for Ethics of Animal Experimentation of the Research Institute of Influenza (Permit Number: 0618). All efforts were made to minimize animal suffering.

## 5. Conclusions

Overall, this work shows that expression of recombinant fusion protein—based on flagellin, the conservative viral antigens, M2e and the 76–130 a.a. fragment of the second subunit of HA—in plants is feasible and could became a promising approach for the development of a plant-produced vaccine against influenza. Modification of the design of the fusion protein could help to enhance the immunogenicity of the HA-derived peptide and increase the overall efficiency of the candidate vaccine.

## Supplementary Materials

The following are available online at https://www.mdpi.com/2223-7747/9/2/162/s1, Figure S1: Western blot of proteins of proteins isolated from *N. benthamiana* plants at 2, 4, 6, and 8 days after agroinfiltration and separated by SDS-PAGE.

## Abbreviations

HA	Hemagglutinin
PVX	Potato virus X
BAL	Broncho-alveolar lavage
