# Supplementary material for: Plant-Produced Recombinant Influenza A Virus Candidate Vaccine Based on Flagellin Linked to Conservative Fragments of M2 Protein and Hemagglutintin
Source: Plants (Basel). 2020 Jan 29;9(2):162. doi: 10.3390/plants9020162 (PMC7076671; doi:10.3390/plants9020162)
Supplement: Supplementary file 1 [file plants-09-00162-s001.pdf]

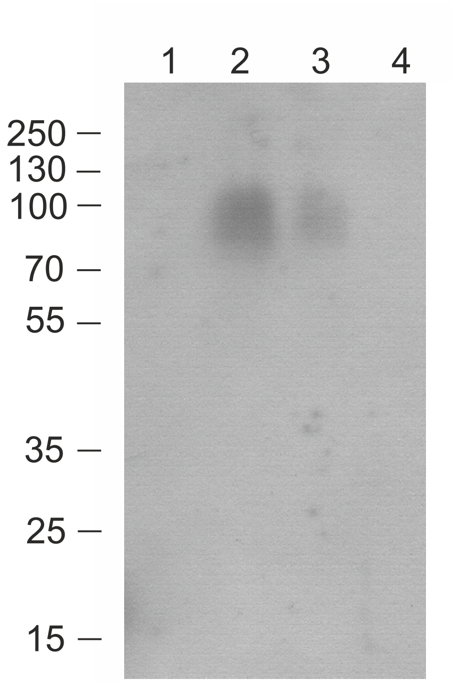

Figure S1. Western blot of proteins isolated from *N. benthamiana* plants at 2, 4, 6, and 8 days after agroinfiltration and separated by SDS-PAGE.

Total protein samples were isolated from leaves infiltrated with vector pEff_Flg4M2eHA2-1 at 2 (lane 1), 4 (lane 2), 6 (lane 3), and 8 (lane 4) days after agroinfiltration.
Positions and sizes of molecular weight markers (kD) are shown to the left of the image.
